# Performance evaluation of the nanoScan^®^ P123S total-body PET

**DOI:** 10.1186/s40658-025-00817-5

**Published:** 2025-12-08

**Authors:** Dániel Réti, Carlos-Alcaide Corral, Islay Cranston, Victoria J. M. Reid, Kerry M. O’Rourke, Timaeus E. F. Morgan, Axel Montagne, Maurits A. Jansen, Valeria K. Burianova, Andrew Sutherland, Péter Major, Kálmán Nagy, Gergő Bagaméry, Adriana A. S. Tavares

**Affiliations:** 1 Mediso Ltd, Laborc Utca 3, Budapest, 1037 Hungary; 2 Centre for Cardiovascular Science, University of Edinburgh, Edinburgh, EH16 4TJ UK; 3 Edinburgh Imaging, University of Edinburgh, Edinburgh, EH16 4TJ UK; 4 Dementia Research Institute, University of Edinburgh, Edinburgh, EH16 4SB UK; 5 Centre for Clinical Brain Sciences, University of Edinburgh, Edinburgh, EH16 4SB UK; 6 Department of Radiology and Medical Imaging, School of Medicine, University of Virginia, Charlottesville, Virginia 22903 USA; 7 School of Chemistry, University of Glasgow, Glasgow, G12 8QQ UK

**Keywords:** PET/CT, Total-body, Instrumentation, NEMA, Performance

## Abstract

**Purpose:**

Before utilising preclinical Position Emission Tomography (PET) systems for biological studies, evaluating their performance is important to better qualify the scanner’s applications. This study aims to assess the performance of the new extended field of view (FOV) nanoScan® PET/CT P123S system, developed for rodent total-body PET applications.

**Methods:**

Scanner resolution, noise equivalent count rate (NECR), sensitivity and image quality were evaluated following NEMA NU-4 2008 protocols. Furthermore, a Derenzo phantom and linearity measurements were conducted. In vivo studies were subsequently carried out to evaluate system performance in biological applications.

**Results:**

The scanner spatial resolution according to the NEMA protocol was 1.4 mm using FBP reconstruction, while with iterative reconstruction it was under 0.7 mm. The NECR peak using a 250‒750 keV energy window was 1805.0 kcps at 93.7 MBq and 880.7 kcps at 88.4 MBq for the mouse-sized and rat-sized phantom respectively. The absolute sensitivity was 10.5%. The standard deviation of the uniform area of the image quality phantom was 1.8%, while the recovery coefficients varied between 0.23 and 1.00. The spill-over ratios were 0.04, and 0.04 in the water and air-filled chambers respectively. Quantitative bias was < 4% with a linear response up to 105 MBq. Total-body rat images were successfully acquired using the new system.

**Conclusion:**

The new extended FOV PET system has improved sensitivity and count rate performance compared with previous systems. Its spatial resolution and quantitative accuracy are well-suited for preclinical PET applications. The extended FOV enables total-body imaging of both mice and rats.

**Supplementary Information:**

The online version contains supplementary material available at 10.1186/s40658-025-00817-5.

## Introduction

Total-body Positron Emission Tomography (PET) imaging is an emerging technique in which the entire body of the subject remains within the field of view throughout the scanning session. By capturing tracer kinetics across the entire body in a single acquisition, total-body PET enables shorter scanning durations, improved temporal resolution, and more precise quantification of radiotracer distribution. The benefits of using this recent generation of scanners in clinical and preclinical research are becoming increasingly evident [1–5]. The primary factors determining whether a scanner can be used for total-body preclinical PET imaging are the field of view (FOV) entirely accommodating the studied animal [6] and the count rate performance allowing to handle the entire injected dose [1, 7].

In 2007, the first widely available commercial preclinical PET/CT system was developed by Siemens [8, 9], which was soon followed in 2009 by Mediso’s nanoPET/CT [10]. Since then, many other manufacturers introduced their own preclinical PET/CT scanner [9, 11–25]. With the introduction of silicon photomultiplier technology, PET/Magnetic Resonance Imaging (MRI) inserts became available [26–31]. In the recent years, preclinical PET system design started to evolve towards longer axial FOV (AFOV) to accommodate the community’s need for total-body imaging. Good example for this is the Si78 PET/CT developed by Bruker, whith 150 mm AFOV [23], or the SimPET XL PET/MRI insert from Brightonix [30], with 110 mm axial coverage. Following this trend, a new PET/CT system was developed by Mediso Ltd.; nanoScan® PET/CT P123S. This system is similar to the nanoScan® PET/CT P122S camera [13, 14], with a key difference of expanding the AFOV from 100 to 151 mm by adding one more detector ring.

The purpose of this study was to evaluate the performance of the nanoScan® PET/CT P123S system and to discuss its suitability for total-body preclinical PET studies. The framework of the performance evaluation was based on the NEMA NU-4 2008 [32]. Since this protocol is widely used, it offers an opportunity to compare the results of this study with previous works [9, 11–31].

## Materials and methods

### Scanner description

**Fig. 1:**
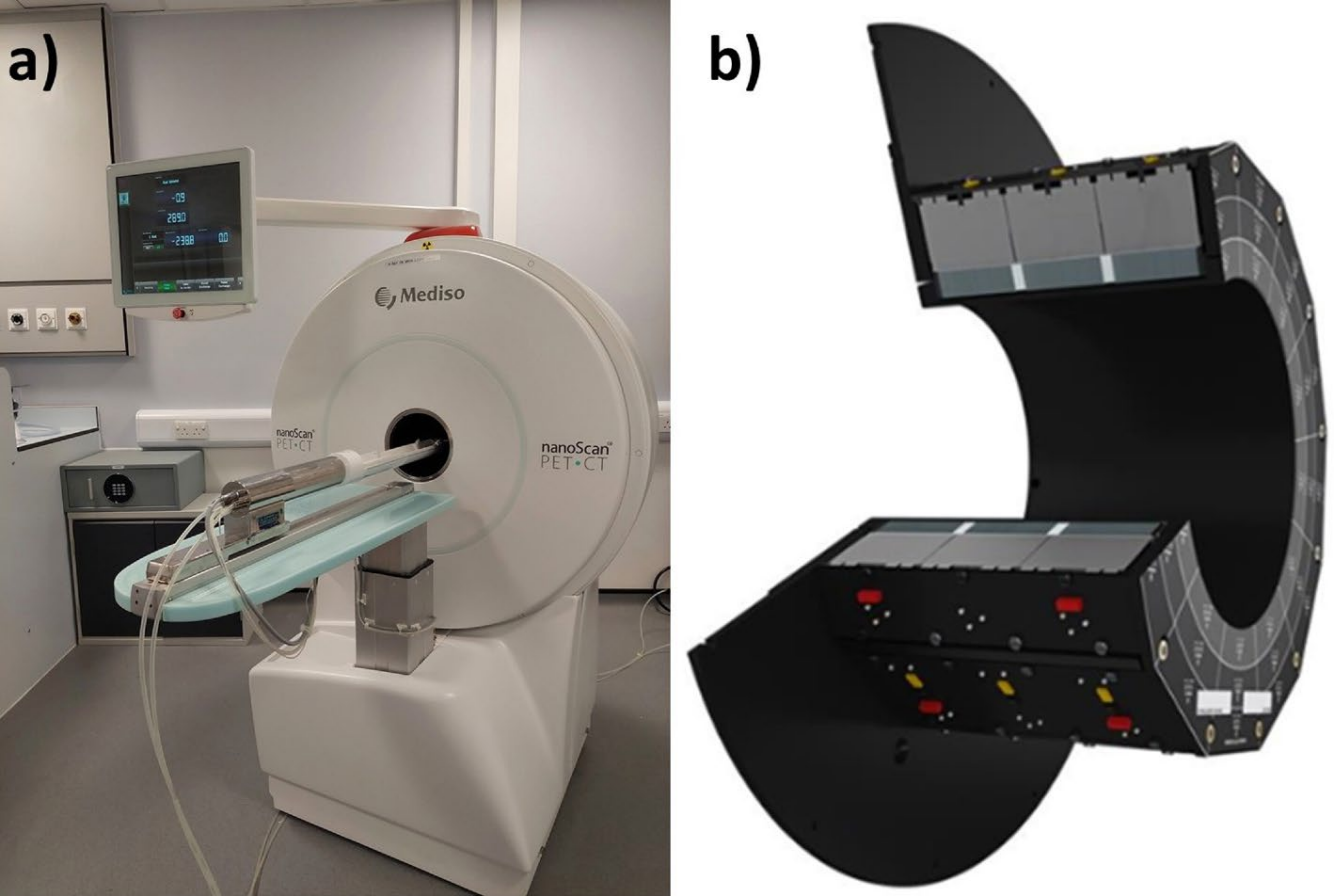
**a** Image of the nanoScan® P123S PET/CT system. **b** The extended FOV PET ring

As mentioned above, the nanoScan® PET/CT P123S is the next generation of the nanoScan® P122S system, which is suited for better total-body PET applications, by extending the AFOV. The PET subsystem features a three-ring detector design, with each ring housing 12 detectors, totalling 39 × 39 Lutetium Oxyorthosilicate (LSO crystal) needles (1.11 × 1.11 × 13 mm) per detector. The bore diameter of the system is 169 mm. It has 120 mm transaxial FOV (TFOV) and 151 mm AFOV. The system is able to collect data in 1:1, 1:3, and 1:5 coincidence modes (Table 1). The CT subsystem offers a 12 cm TFOV and a 45 cm helical range. There are different options to generate material maps from CT acquisition, the simplest model is the so-called "water–air" map, which is binary. The system is able to move the bed in axial and vertical directions. Animal monitoring was also available. The image of the PET/CT scanner, and the PET ring is in Fig. 1.

**Table 1:** List of the available acquisition and reconstruction parameters, and their trade-offs

Acquisition		
parameters	Options	Explanation
Coincidence mode	1:1	Higher coincidence numbers mean larger TFOV, larger data size, and slower reconstruction
	1:3	
	1:5	
Binning/Rebinning		
Parameters	Options	Explanation
Energy window	400–600 keV	Smaller energy window provides better contrast (general setup), larger means higher sensitivity
	250–750 keV	
Ring difference (2D)	13–129	Lower ring difference means better axial resolution and contrast, but lower sensitivity
Smooth rebinning	On	Resolution enhancing deconvolution method in sinogram space based on smoothstep function
	Off	
Reconstruction		
Parameters	Options	Explanation
Voxel size	0.1 mm(2D), 0.2 mm(3D) -…	Smaller voxel size means better resolution, slower reconstruction, and worse uniformity
Iteration number	1-…	Higher iteration number means slower reconstruction, but better convergence
Subset number	1–6	Higher subset means faster reconstruction, but worse uniformity
Regularisation	None	Higher regularisation means better uniformity but worse resolution
	Low	
	Medium	
	High	
Edge artefact reduction	On	It decreases overshooting at sharp activity density steps
	Off	
Spike filter	On	It eliminates outlier voxel values; with point source measurements it should be switched off
	Off

### Study design

The bedrock of the methodology of this study was the NEMA NU-4 2008 guidelines [32], expanded with additional reconstructions and energy windows to further investigate the system capabilities. The rationale behind constructing the study such way was to quantify the performance metrics, while enabling comparison with other systems using previously established techniques.

For NEMA spatial resolution measurements, a sodium-22 (Na-22, Eckert and Ziegler, Germany) point source (0.57 MBq) and Derenzo phantom with varying rod sizes of 0.7, 0.8, 0.9, 1.1 and 1.2 mm was used. For the scatter fraction and count rate measurements, the standard NEMA mouse and rat-sized phantom with body made of high-density polyethylene (0.96 ± 0.1 g/cm^3^) was filled with radioactivity [32]. The sensitivity measurements required a Na-22 point source (Eckert and Ziegler, Germany, 0.39 MBq). To assess the image quality, the standard image quality NEMA phantom [32] was used. A cylindrical phantom with a diameter of 6 cm and length of 20 cm was filled with activity for linearity tests. All phantoms, except the point sources, were produced by Mediso Ltd. Following completion of scanner performance analysis, in vivo PET imaging of rats was carried out.

Acquisition data was reconstructed with the manufacturer’s software, Nucline®. The reconstruction parameters were harmonised, standardised, and optimised (Supplementary Fig. 1–5) for use, and they were used throughout the study, except for scanner resolution measurements, as these aimed to access only one aspect of the system performance. The trade-offs of PET imaging parameters are in Table 1. All data in the study was reconstructed with 1:5 coincidence mode, random, dead-time, attenuation and scatter correction, unless the NEMA guidelines instructed otherwise.

### Spatial resolution

The quantification of the spatial resolution of the system was performed by following the NEMA NU-4 2008 protocol [32], and by employing the Derenzo phantom.

The Na-22 source was positioned at the centre of the FOV and radially moved by the scanner bed by 5 mm increments. Four-minute acquisitions were taken at the axial centre and one-fourth of the AFOV.

Two reconstruction techniques were used for the point source; filtered back projection (FBP) to comply with the standards, and iterative since many other performance papers used that [11, 13, 16, 20–22, 24, 26–31]. For the FBP reconstruction, data were rebinned using the Fourier Rebinning (FORE) algorithm with Ramlak filter. The ring difference was the smallest available of 13, while the voxel size was 0.1 mm, the smallest available for 2D reconstructions. Data was processed with Mediso’s Tera-Tomo™ 3D PET iterative reconstruction. While considering the effect of each parameter, 10 iteration, 1 subset, no regularisation, and 0.2 mm voxel size, which is the minimum for 3D reconstruction was employed. The radial, tangential and axial profiles of the images were examined, and the full width at half maximum (FWHM) and full width at tenth maximum (FWTM) of the peaks were calculated using the Mediso Image Quality Center software.

The Derenzo phantom was filled with 7.5 MBq of [^18^F]NaF, and the counts were collected for half hour. The image was then reconstructed using Tera-Tomo™ 3D iterative reconstruction, with similar parameter set than used for the point source data, except the iteration number, which was raised to 120 due to the more complex radioactivity distribution.

### Scatter fraction and count rate measurements

The aim of this measurement is to examine the ‘relative system sensitivity to scattered radiation’, and to quantify ‘the effects of system dead-time and the generation of random coincidence events’ [32].

The phantoms were filled with 120.1 ± 0.06 MBq of [^18^F]NaF and data were collected for one minute every 10 min over a period of 16 h. The scanner detector crystals are lutetium-based; therefore a 20-min data were collected for background correction.

Data were rebinned using single slice rebinning (SSRB) without applying any corrections with a 4 ns coincidence window. NEMA guidelines were followed to calculate the total, true, random, and scattered rates, and the scattered fractions from the sinograms at each time points. Random rates were calculated using delayed window.

### Sensitivity

The aim of this performance assessment step was to measure the ability of the scanner to detect radiation used for PET image formation.

The Na-22 point source was placed in the centre of the FOV, and moved along the axis in 0.58 mm (half the crystal pitch) steps. In each position data was acquired for 3 min. 20-min background data was utilised here as well.

Collected data were rebinned using SSRB with ring difference of 129. Then, by following the NEMA guidelines, sensitivity and absolute sensitivity was calculated at each point.

### Image quality

The purpose of this measurement was to quantify the system ability to tackle the challenges present during in vivo imaging, like the presence of hot and cold lesions, uniform areas, and small anatomical structures.

The phantom was filled with 3.7 MBq of [^18^F]NaF at the beginning of the experiment, and 20-min acquisition was performed. CT imaging was done on the phantom to create the binary material map for the reconstruction. The CT acquired zigzag trajectories, with 360 projections. Tube voltage was 50 kV, while the tube current was 980µA. FBP with Cosine window was used to reconstruct the CT image.

The reconstruction parameters were carefully optimised (Supplementary Figs. 1–5) and the following parameters were considered suitable for subsequent linearity and animal imaging studies: Tera-Tomo™ 3D iterative reconstruction with 10 iterations, 6 subsets, 0.3 mm voxel size, and high regularisation. Edge artifact reduction and spike filter were used. Then, by using the manufacturer’s Mediso Image Quality Center software the uniformity, recovery coefficient, and spill-over ratios were calculated in accordance to the NEMA guidelines [32].

### Linearity

To investigate the scanner quantitative accuracy across a wide range of activity levels, linearity experiments were carried out.

The phantom contained 105 MBq of [^18^F]NaF at the beginning of the acquisition. In the first hour, the acquisition time was 6 min with a 10-min wait, this gradually increasing to 20 min of acquisition with a 20-min wait by the fourth hour. The total acquisition time was 9 h and 44 min. The CT scan parameters were the same as they were in the image quality phantom measurements except the trajectory here was helical, with pitch number 1.

The images were reconstructed with both 3D Tera-Tomo™ iterative and FBP. The iterative reconstruction was the same optimised protocol from the image quality measurement. For the FBP method, the rebinning was performed by using SSRB algorithm, and Ramlak filter. To enhance the smoothness of the image the highest ring difference of 129 was used, while to improve uniformity the voxel size was set to be 0.7 mm. ‘Smooth rebinning’ option was turned on.

The volumes of interest were placed in the phantoms, and the average concentration of this area at each time point were measured using the InterView™ Fusion software version 3.11 (Mediso Ltd, Hungary). The initial activity of the phantom was measured with the calibrator (Capintec, CRC–25R, USA). Calculated activity was computed considering the radioactive decay. The calculated versus measured activity of the whole phantom was plotted to get the linearity graphs and was examined by linear regression. The accuracy of each data point was calculated by dividing the measured activity with the theoretically calculated activity.

### Animal experiment

In order to demonstrate the system ability to image large rodents, and to illustrate its resolution, in vivo measurements are presented.

That in vivo data in this paper were generated as part of an ongoing longitudinal experiment. A single male Sprague Dawley rat was used and scanned under general anaesthesia at different ages, and weights: two-week-old at 30.6 g (10 MBq) and ten-week-old at 361 g (12.1 MBq), and six-months-old with at 703 g (23.8 MBq). The animal was housed under standard light dark conditions (12:12 h) with food and water available ad libitum. The animal bed was heated to 37 °C, while the breathing and temperature of the animal was monitored. During the measurements the ARRIVE guidelines were followed. The injected tracer was [^18^F]SynVesT-1 [33] with molar activities ranging between 47.6 and 108 GBq µmol^−1^ with > 95% radiochemical purity. All the data were collected for 90 min. CT scanning of the animal was performed with the same parameters as presented in the image quality phantom section. The estimated dose (CTDI_volume_) per scan was 30.6 mGy.

PET data of the two-, and ten-week-old rats was reconstructed utilising the standardised parameter set from the image quality measurement. The reconstruction was performed between 30 and 60 min after the injection. Furthermore, the brain of the six-month-old rat was also reconstructed using the same high-resolution protocol used in the performance assessment experiments carried out with the Derenzo phantom. The aim of this reconstruction was to illustrate that the system is capable of producing images with great detail, which is an aim very similar to the one of the Derenzo phantom measurement.

Standardised uptake value (SUV) images were generated by normalising radioactive concentration for animal body weight and injected activity. 3D Maximum Intensity Projection (MIP) images were also generated using PMOD software version 3.7 (PMOD Technologies, Switzerland). The high resolution brain PET SUV image was filtered using a Gaussian filter with a kernel of 1 × 1 × 1 mm, then it was co-registered to the Waxholm rat brain MRI atlas [34] using PMOD.

## Results

### Spatial resolution

**Fig. 2:**
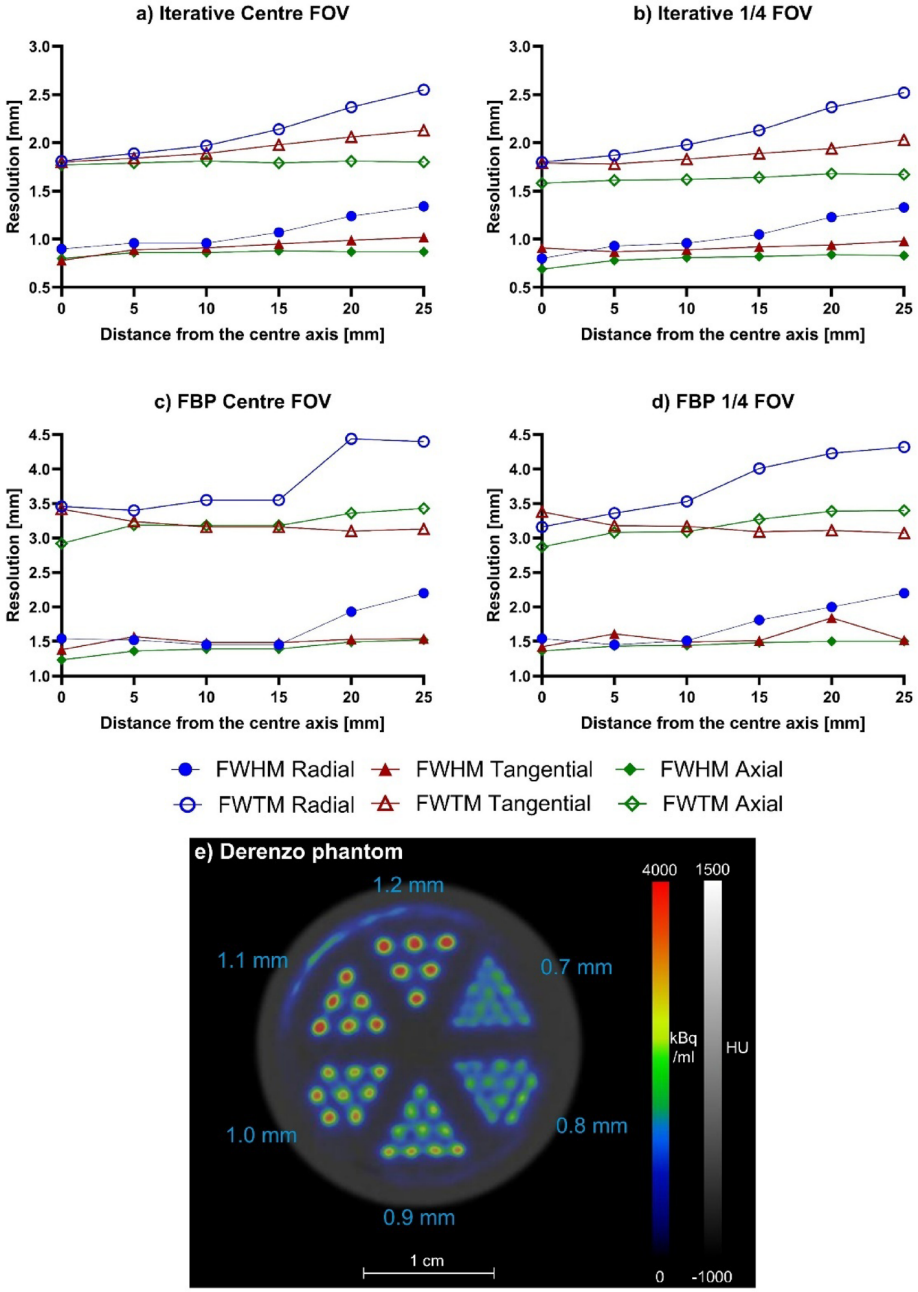
Spatial resolution of the nanoScan® P123S PET system using the Na-22 point source. The source was in the centre of the AFOV reconstructed with iterative method (0.2 mm voxel size) (**a)** and FBP (0.1 mm voxel size, 0.585 mm slices) (**c)**, and in the one-fourth of the AFOV reconstructed with iterative method **(b)** and FBP (**d)**. Image **(e)** shows an axial slice of the Derenzo phantom

The average FWHM value of the three axes of the Na-22 point source was 0.6 mm in the centre when using iterative methods of reconstruction (Fig. 2A) and 1.4 mm when using FBP reconstruction (Fig. 2C). The 0.7 mm rods in the Derenzo phantom PET image were distinguishable (Fig. 2E).

### Scatter fraction and count rate measurements

**Fig. 3:**
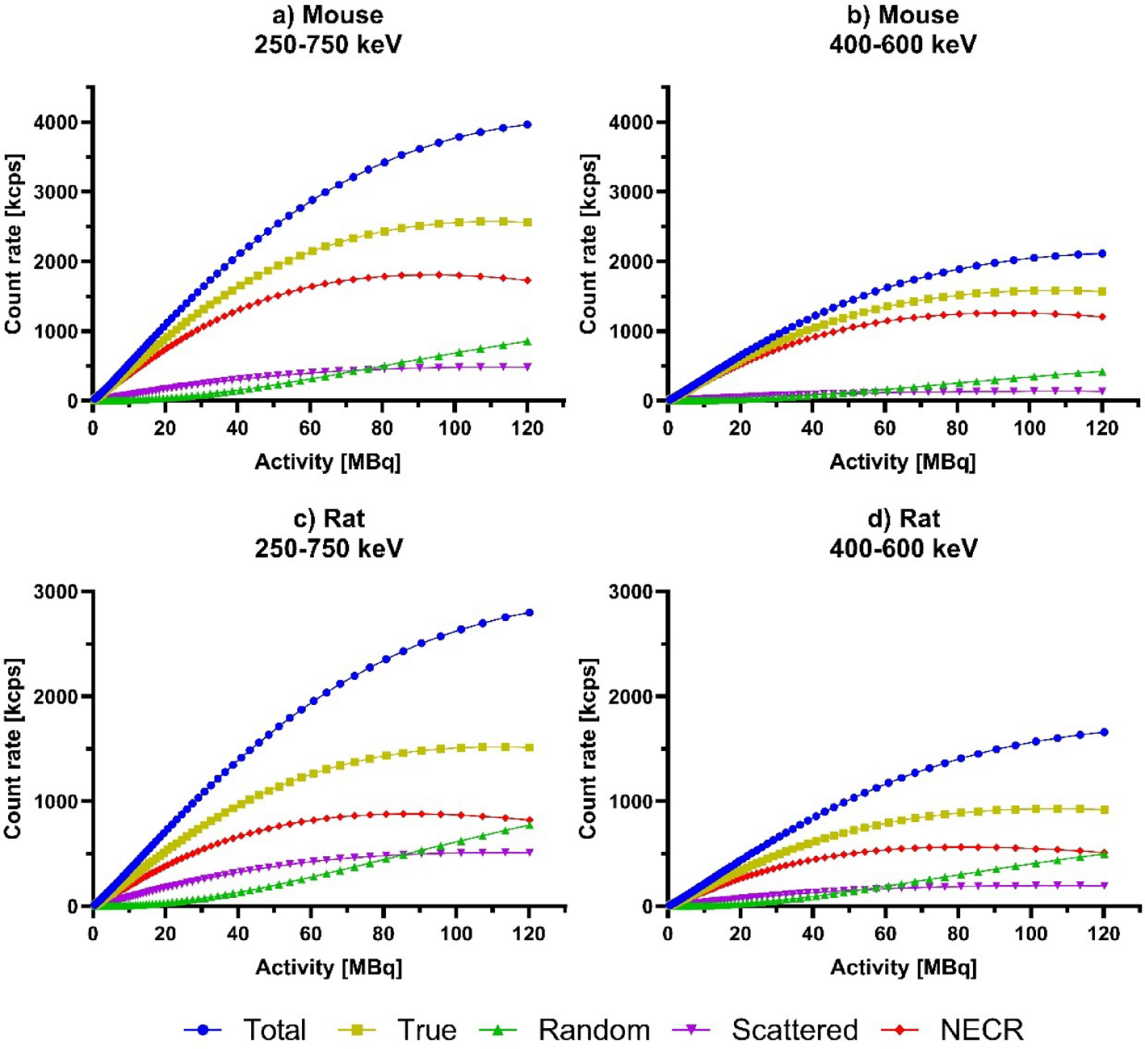
Count rates measured for the nanoScan® P123S PET/CT system. The results of the mouse-sized phantom with 250‒750 keV window **(a)** and with 400‒600 keV window **(b)**. The rat-sized phantom with 250‒750 keV window **(c)**, and with 400‒600 keV window **(d)**

The scatter fraction and count rate results are summarised in Table 2. In the mouse phantom, the peak NECR value was 1805.03 kcps at 93.7 MBq with 0.1615 scattered fraction, while in the rat phantom the NECR peak was 880.71 kcps at 88.4 MBq with scatter fraction 0.2528 (Fig. 3).

**Table 2:** Summary of the results of the scatter fraction and count rate measurements

Phantom	Energy window	NEC peak [kcps]	Activity of the peak [MBq]	SF
Mouse	250‒750 keV	1805.03	93.72	0.1615
Mouse	400‒600 keV	1257.13	94.21	0.0788
Rat	250‒750 keV	880.71	88.4	0.2528
Rat	400‒600 keV	563.6	81.83	0.1746

### Sensitivity

**Fig. 4:**
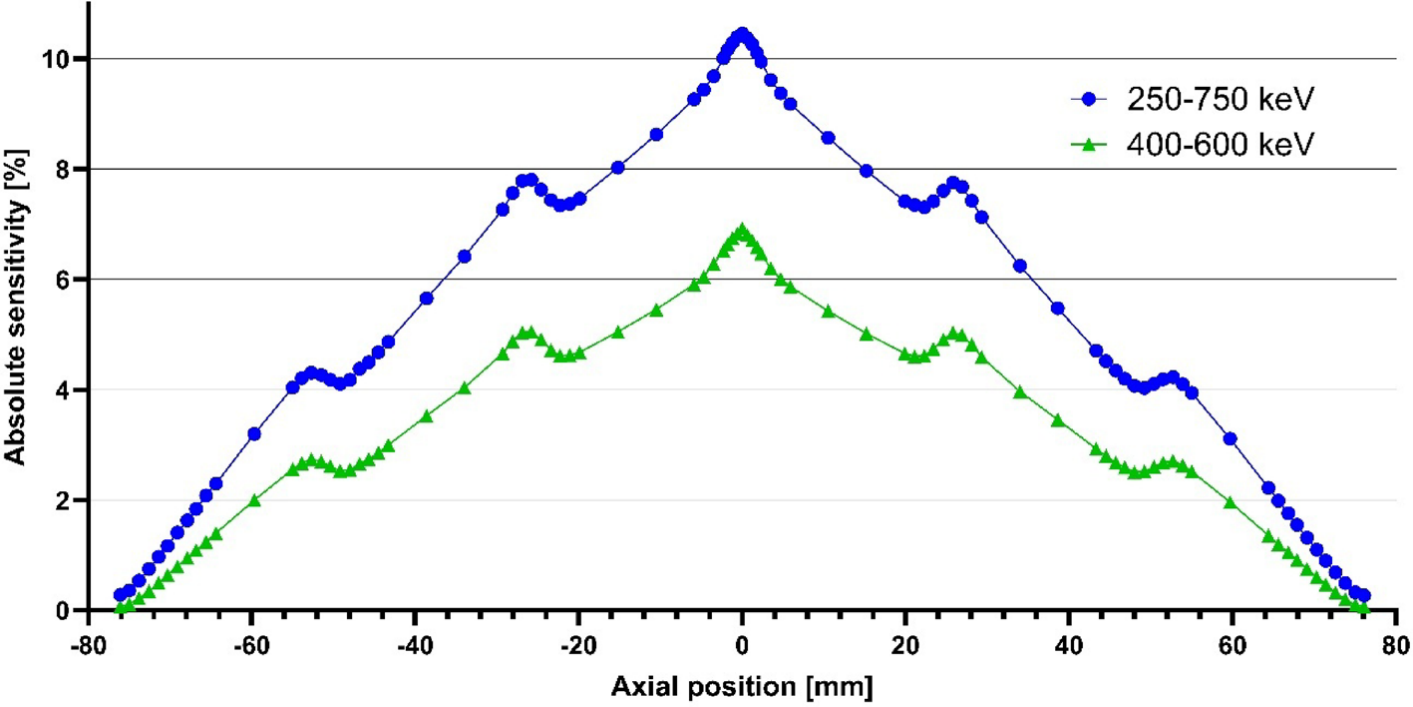
The absolute sensitivity profiles of the nanoScan® P123S. Observed transition points are due to known gaps in the detector ring system

The peak and absolute sensitivity was 94.4 cps/kBq and 10.46% with the 250‒750 keV window, while with 400‒600 keV window it was 62.2 cps/kBq and 6.93%. The absolute sensitivity profiles are presented in Fig. 4.

### Image quality

**Fig. 5:**
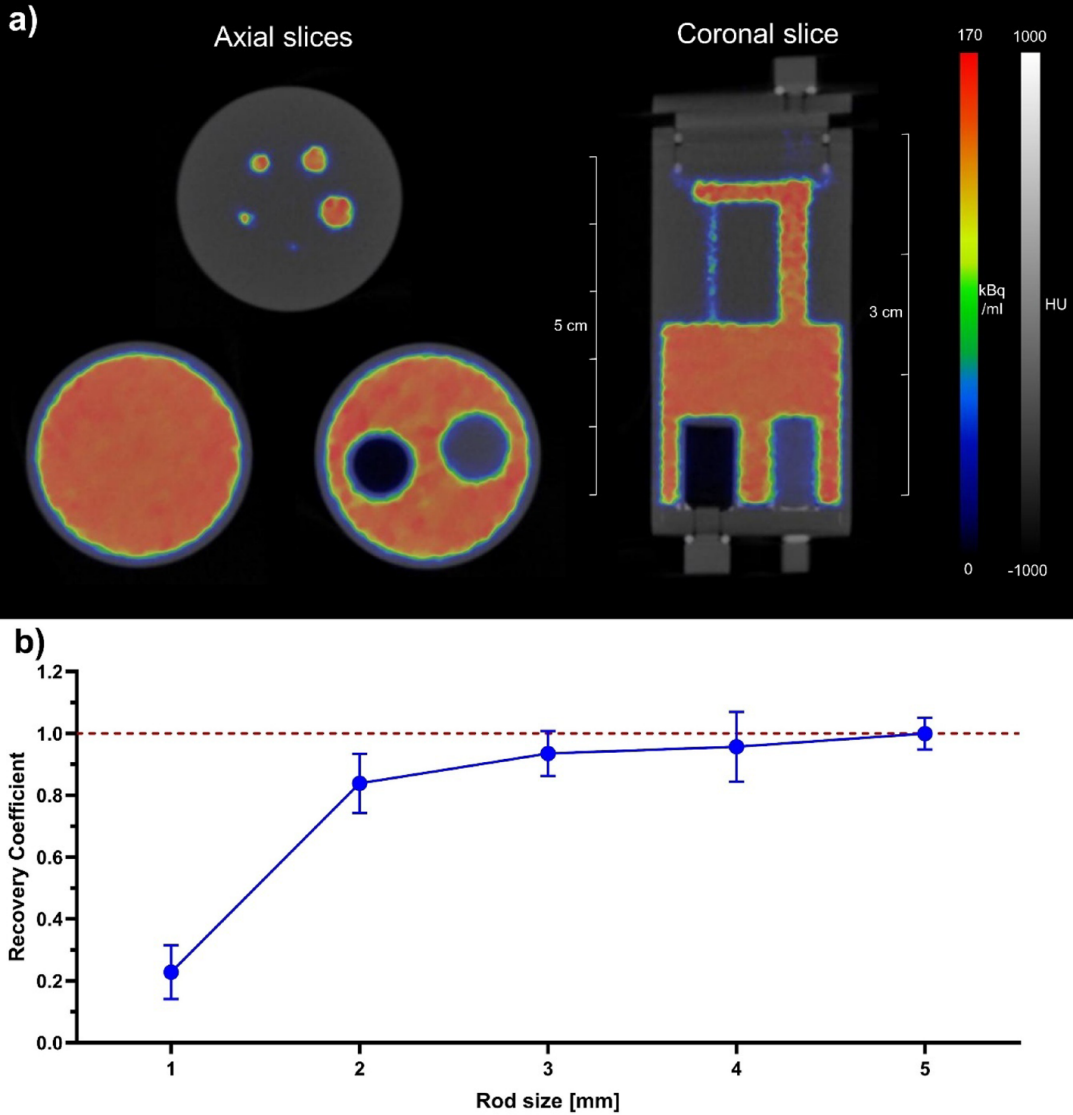
**a** Representative image of the NEMA NU-4 image quality phantom. 3D TerraTomo® PET reconstruction was used. **b** The calculated recovery coefficients from the same image plotted against the rod sizes

In the uniformity area of the image quality phantom **(**Fig. 5**)**, the average concentration was 153.06 kBq/mL, which meant 1.8% standard deviation. The maximum value was 168.14 kBq/mL, while the minimum was 140.39 kBq/mL. The recovery coefficients and their standard deviations from smallest to largest ended up being 0.22 (38.1%), 0.84 (11.4%), 0.93 (7.7%), 0.96 (11.82%), and 1.00 (7.8%). The spill-over ratios were 0.04 (17.9%) in the water-filled chamber and 0.04 (13.8%) in the air-filled chamber.

### Linearity

**Fig. 6:**
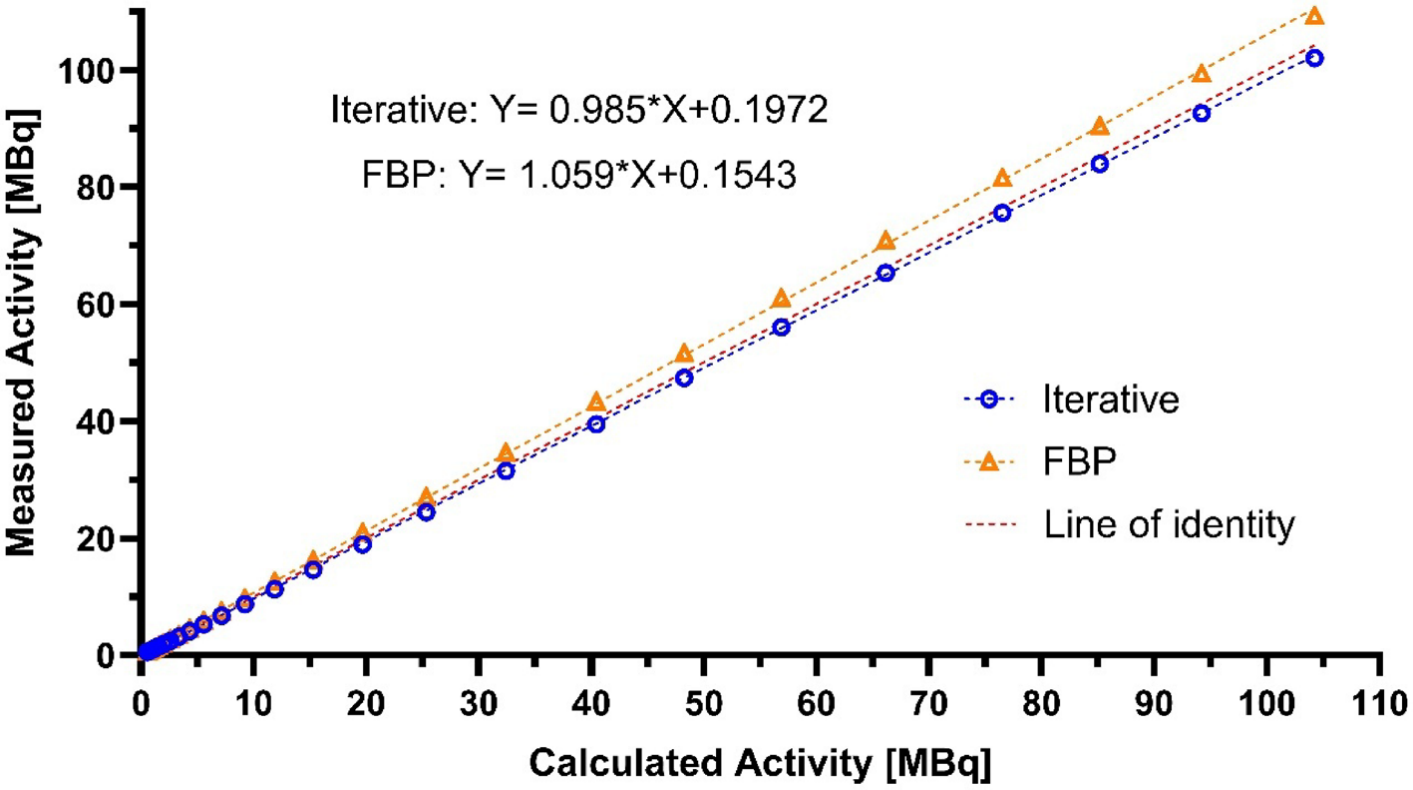
The linearity of a cylindrical phantom with two different reconstruction methods. Linear interpolation was used on both datasets

The range of the activity used for linearity experiments was 2.5–105 MBq **(**Fig. 6**)**. The mean bias of the iterative reconstruction methods was − 3.54% with 1.78% standard deviation, while the mean was 6.01 with 0.97% standard deviation in the case of the FBP reconstruction.

### Animal experiment

**Fig. 7:**
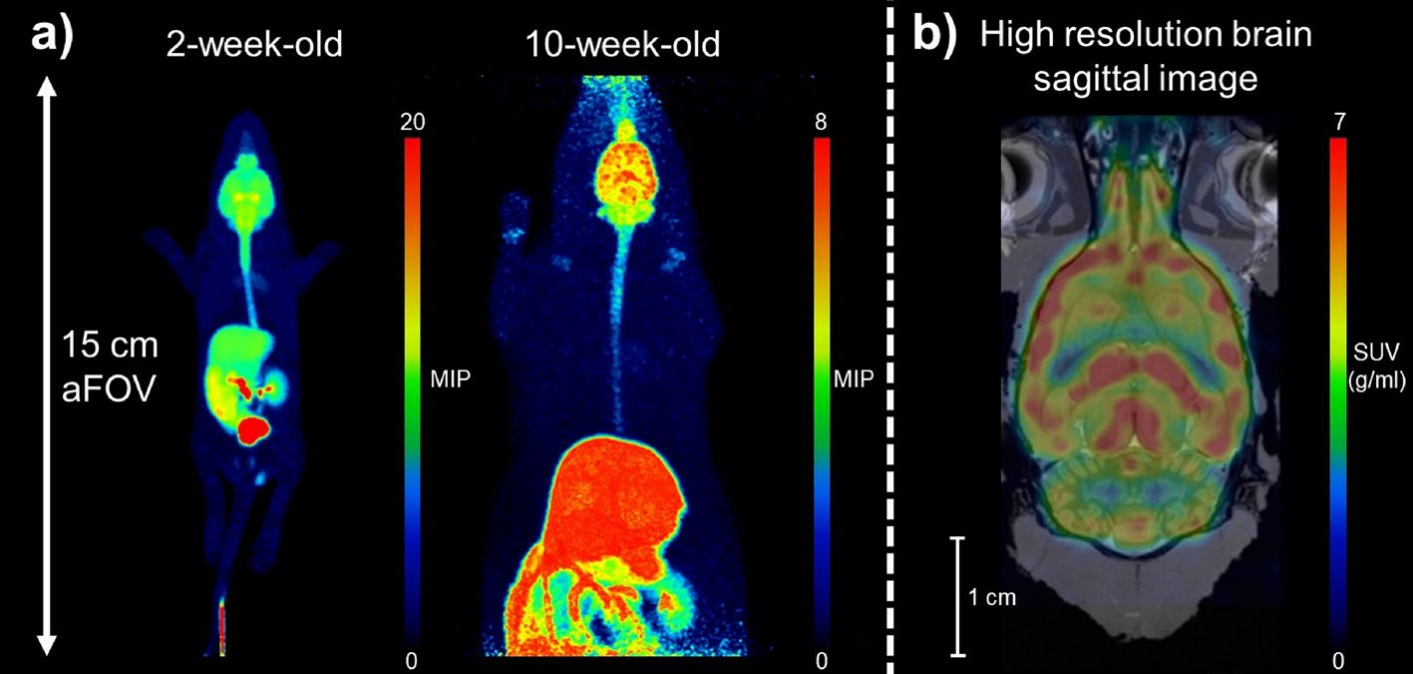
Demonstrative animal images from the scanner, collected following intravenous bolus injection of [^18^F]SynVesT-1, a PET radiotracer used for imaging synapses in the brain. **a** Maximum intensity total-body projections of PET/CT images of a 2-week-old (30.6 g, 10 MBq), and ten-week-old (361 g, 12.1 MBq) rat. **b** A high resolution, SUV weighted, and smoothed sagittal slice of the brain of a 6-month-old (703 g, 23.8 MBq) rat overlayed on the Waxholm space atlas

In vivo PET/CT imaging studies of rats over three different ageing time points produced good quality data and coverage of the whole-body up to ten weeks (Fig. 7A). This illustrates the scanner’s ability to scan rats using total-body PET. The scanner’s ability to enable high resolution imaging of the adult rat brain is illustrated in Fig. 7B.

## Discussion

The aim of this study was to evaluate the performance of a total-body preclinical PET scanner and to explore its capability for conducting total-body PET studies in vivo.

A well performing total-body PET system requires both high sensitivity and ability to handle high count rates [1, 7]. These parameters showed improvement compared to the previous generation of nanoScan® systems [13, 14], with absolute sensitivity increasing from to 7.66 to 10.46%, and the mouse NECR peak demonstrating a 1042 kcps improvement.

The absolute sensitivity peak was 10.46%, which, when compared with other scanners with similar geometry [9, 11–15, 17–20, 22–24], is among the highest, when comparing with results with similar energy windows. The PET ring successfully manages high activities quantitatively, as evidenced by the absence of saturation in the total count curves up to 120 MBq, and the linear period of the NECR curves extend to 40‒50 MBq. The NECR peaks are the highest among all the preclinical PET scanners previously reported [9, 11–31]. The activity levels of the NECR peaks were high (Table 2), and linear response was acquired over a wide activity level (Fig. 6). These results proves that the system is capable handling very high activity levels.

The resolution of the system was similar to the resolution of other Mediso PET systems [13, 14], due to the similar crystal matrix and diameter [35].

The image quality phantom results demonstrate excellent performance compared to other preclinical PET scanners, with one of the lowest standard deviations of the uniform area (1.8%), rapid recovery of radioactivity from small (1 mm) to large (5 mm) rods, and low SOR values [9, 11–31].

The linearity measurement showed that the quantitative accuracy of the scanner is reliable within a wide range of activities (2.5–105 MBq). This result suggests that the system is suitable for kinetic studies with external blood sampling by beta probe technology, as these studies require relatively high activity levels to ensure accurate detection of blood radioactive concentrations by the beta probes (known to have lower sensitivity versus PET scanner). The difference between the two reconstruction techniques may be due to differences in the corrections applied during the reconstruction process. Notwithstanding, for both FBP and interactive methods, the measured bias was < 10%, suggesting both methods can robustly provide quantitative PET data.

The in vivo measurements demonstrated that the 151 mm field of view is sufficient to cover the whole body of a mouse or a young rat, and opens possibilities to investigate multi-organ connectivity. A good example is the kinetic analysis of the brain and gut, which can be very useful in Parkinson disease research [36]. There is also evidence that the vena cava*,* which is included in a single FOV even in the case of large rat, might be a better image derived input function reference point than the heart [37], thereby facilitating kinetic modelling efforts when using image-derived input functions. Furthermore, the new system has high NECR peak and high sensitivity, which increase PET data statistical quality. Owing to these two factors the temporal resolution of an animal scan can be greater than other preclinical systems, allowing more detailed kinetic analysis.

Table 3 presents the performance evaluation of the PET subsystem of various commercial preclinical PET systems [17, 20–23] dedicated to rodent imaging, selected based on commercial availability at the time of publication or extensive prior use in preclinical PET studies [9]. No PET/MRI insert systems were considered when creating the table. Performance metrics are derived from NEMA NU-4 2008 evaluations, also including the best published resolution achieved using the vendor's iterative reconstruction. Table 3 shows that the nanoScan® system performs well in terms of resolution, count rate tolerance, quantitative accuracy and sensitivity, while providing the largest FOV in the market at present.

**Table 3:** Summary of results of published performance evaluation of commercial preclinical systems. In the case of two scanners, the image quality data was originated from different sources; [9]* and [38]**

Name of the system			nanoScan® P123S	Inveon (legacy)	Si78	β-CUBE	SuperArgus 4R	IRIS	MRS Clip-on
Vendor			Mediso	Siemens	Bruker	MOLECUBES /Bruker	Sedecal	Inviscan	MR Solutions
Axial FOV			150 mm	127 mm	150 mm	130 mm	100 mm	95 mm	50.4 mm
Transaxial FOV			120 mm	100 mm	80 mm	72 mm	120 mm	80 mm	67 mm
Resolution using iterative reconstruction			0.7 mm	1.0 mm	0.9 mm	1.2 mm	1.2 mm	0.85 mm	0.7 mm
NECR rate peak [kcps]	Mouse-sized phantom		1805	1758	N/A	300	N/A	185	61.9
	Rat-sized phantom		881	616	N/A	160	N/A	40	16.4
Peak NECR position [MBq]	Mouse-sized phantom		93.7	114	N/A	33	N/A	14	14.9
	Rat-sized phantom		88.4	77	N/A	33	N/A	10	14.4
Absolute NEMA sensitivity (%)			10.46	10.30	9.00	12.40	9.50	8	4.7
Energy window (used for count rate measurement and sensitivity)			250–750 keV	250–750 keV	358–664 keV	255–765 keV	100–700 keV	250–750 keV	250–750 keV
Image quality	Uniformity (%)		1.84	5.29	9.60	7.43	5.70	7	6.3
	Recovery coefficient	1	0.23	0.17	0.04	0.3	0.29	0.14	0.13
		2	0.84	0.48	0.68	0.65	0.9	0.58	0.55
		3	0.93	0.72	0.88	0.95	0.91	0.73	0.84
		4	0.96	0.84	1	0.85	0.95	0.82	0.98
		5	1.00	0.93	0.96	0.95	0.96	0.89	0.96
	SOR	Water	0.04	0.017	0.25	0.08	N/A	0.11	0.07
		Water Std	17.86%	N/A	23.00%	N/A	N/A	16%	12.6%
		Air	0.04	-0.006	0.17	0.08	N/A	0.11	0.17
		Air Std	13.80%	N/A	35.00%	N/A	N/A	11%	10.7%
References			This paper	[8]* [9]	[23]	[21]	[17] [38]**	[20]	[22]

## Conclusions

The nanoScan® PET/CT P123S, featuring an extended FOV PET subsystem, demonstrates enhanced sensitivity and count rate. Extended FOV supports total-body imaging of mice and young rats, while offering broader coverage for aged rats. As detailed in the scanner comparison presented earlier, the nanoScan® exhibited excellent spatial resolution, count rate, and sensitivity. These combined characteristics, along with high spatial resolution and quantitative accuracy, establish the nanoScan® total-body PET/CT as a powerful tool for a wide range of preclinical PET applications.

## Supplementary Information

Additional file1 (DOCX 886 kb)

## Data Availability

Upon request the corresponding author will share any data from the study.
