# Supplementary material for: Performance evaluation of the nanoScan® P123S total-body PET
Source: EJNMMI Phys. 2025 Dec 8;13:2. doi: 10.1186/s40658-025-00817-5 (PMC12779859; doi:10.1186/s40658-025-00817-5)
Supplement: Supplementary file 1 — Additional file1 (DOCX 886 kb) [file 40658_2025_817_MOESM1_ESM.docx]

# Supplementary Data


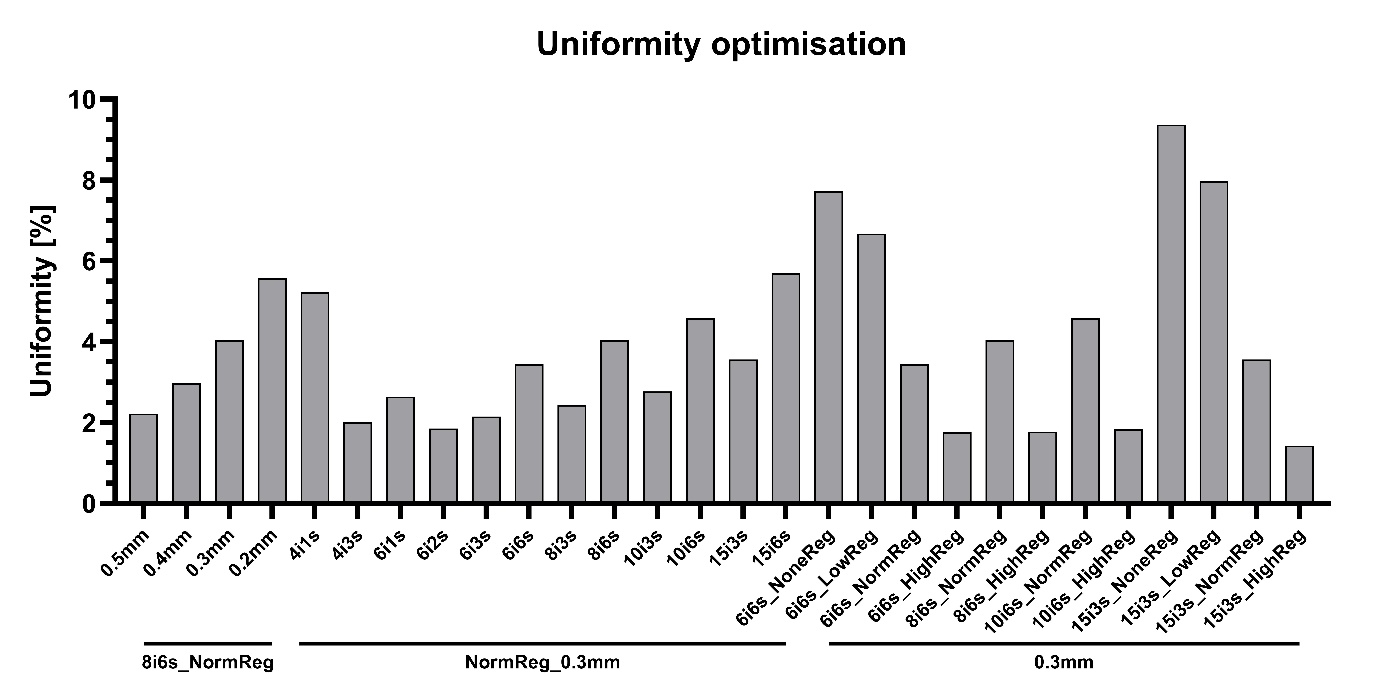


**Supplementary Figure 1.**  Uniformity results obtained using different reconstruction methods.


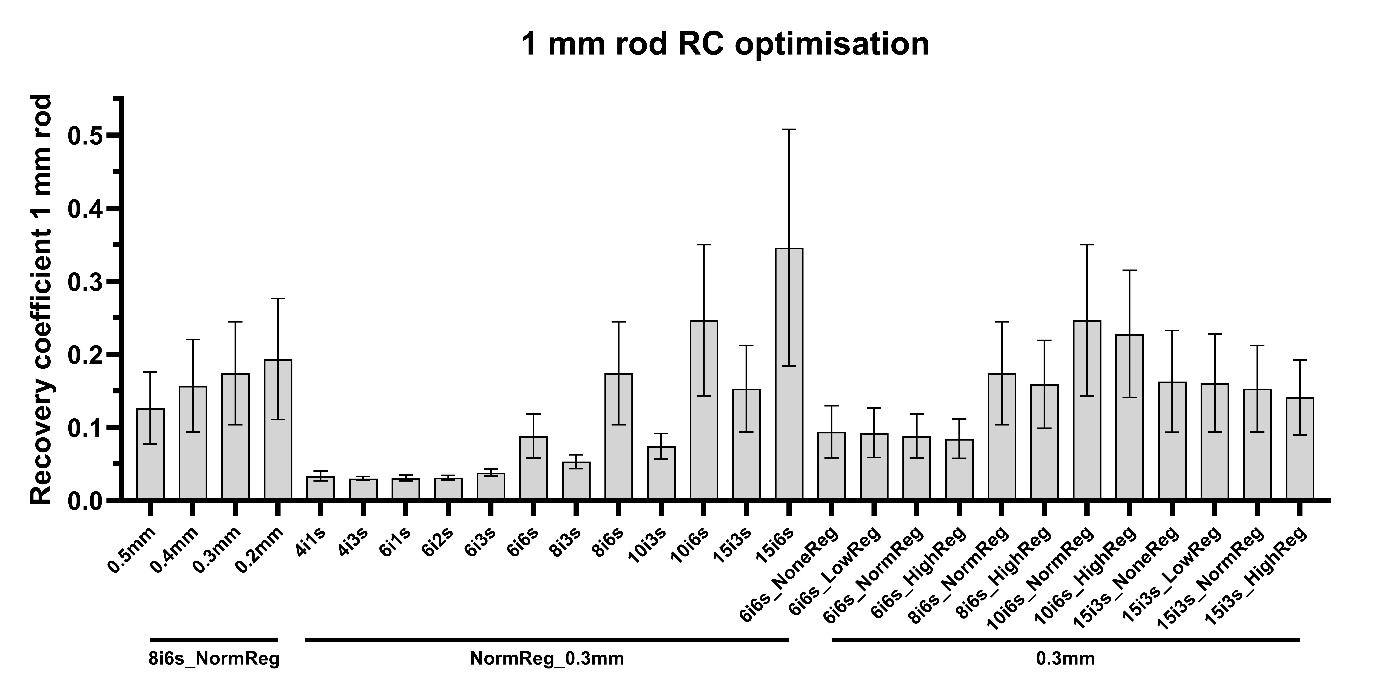


**Supplementary Figure 2.** Recovery coefficient (RC) results in the 1 mm rod for different reconstruction methods.


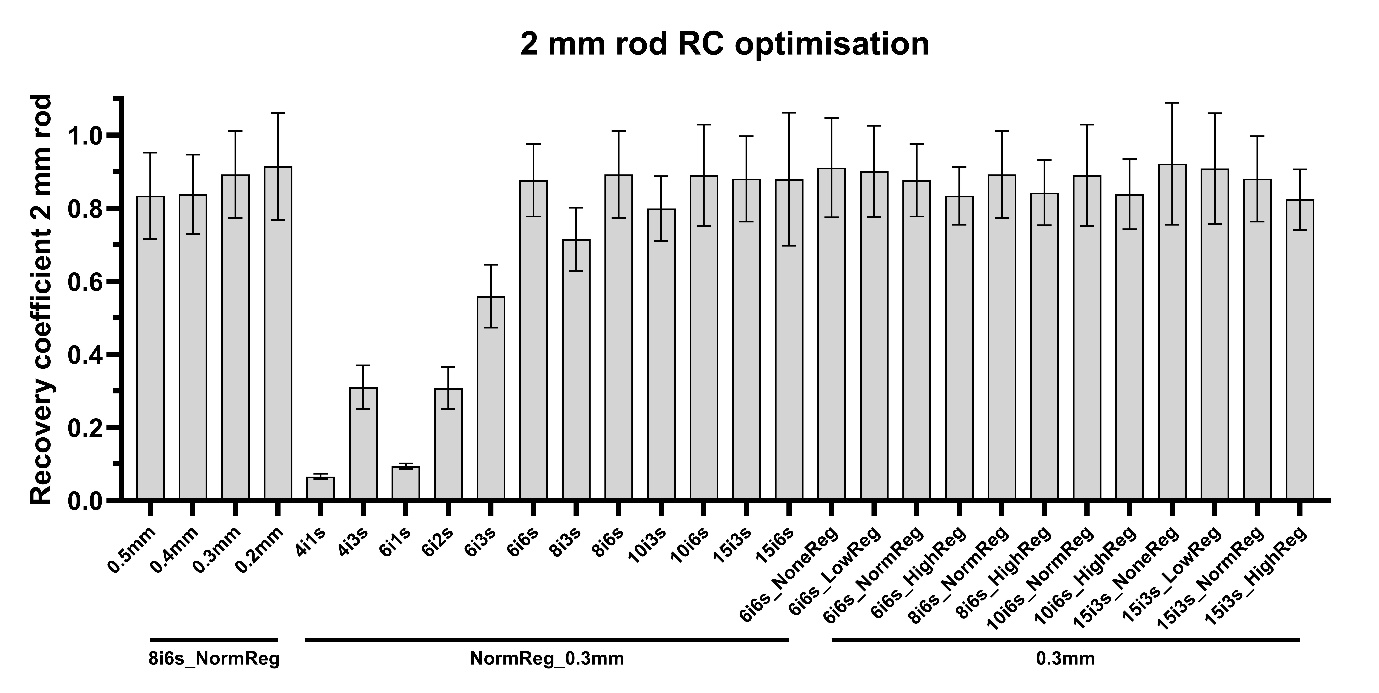


**Supplementary Figure 3.** Recovery coefficient (RC) results in the 2 mm rod for different reconstruction methods.


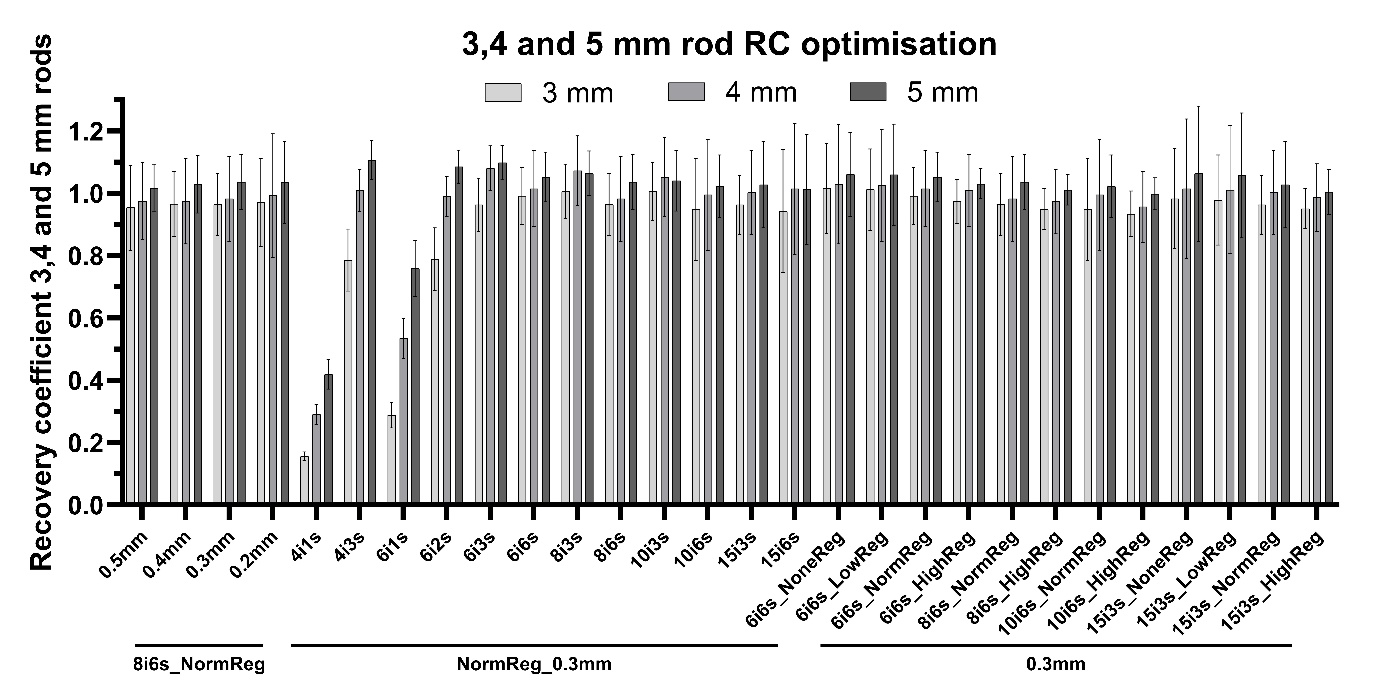


**Supplementary Figure 4.** Recovery coefficient (RC) results in the 3, 4 and 5 mm rod for different reconstruction methods.


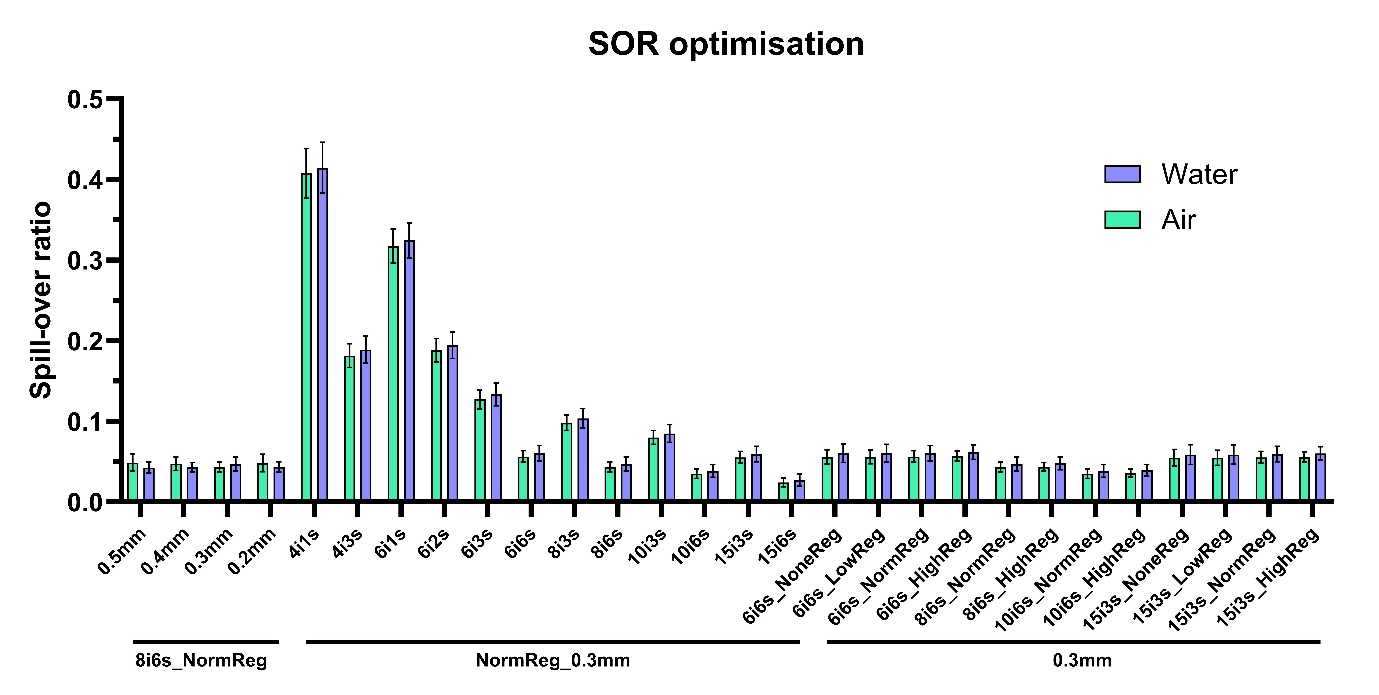


**Supplementary Figure 5.** The spill over ratios (SOR) of the water and air-filled chambers measured using different reconstruction protocols.
